# Inflammatory Response of Ischemic Tolerance in Circulating Plasma: Preconditioning-Induced by Transient Ischemic Attack (TIA) Phenomena in Acute Ischemia Patients (AIS)

**DOI:** 10.3389/fneur.2020.552470

**Published:** 2020-10-29

**Authors:** Laura Colàs-Campàs, Joan Farre, Gerard Mauri-Capdevila, Jessica Molina-Seguín, Núria Aymerich, Ángel Ois, Jaume Roquer, Silvia Tur, María del Carmen García-Carreira, Joan Martí-Fàbregas, Antonio Cruz-Culebras, Tomás Segura, Gloria Arque, Francisco Purroy

**Affiliations:** ^1^Clinical Neurosciences Group, Institut de Recerca Biomèdica de Lleida, Lleida, Spain; ^2^Medical Laboratory, Hospital Universitari Arnau de Vilanova, Lleida, Spain; ^3^Stroke Unit, Department of Neurology, Hospital Universitari Arnau de Vilanova, Lleida, Spain; ^4^Complejo Hospitalario de Navarra, Pamplona, Spain; ^5^Hospital del Mar, Barcelona, Spain; ^6^Hospital Son Espases, Palma de Mallorca, Spain; ^7^Corporació Sanitària Parc Taulí, Sabadell, Spain; ^8^Hospital de la Santa Creu i Sant Pau, Barcelona, Spain; ^9^Hospital Universitario Ramón y Cajal, Madrid, Spain; ^10^Complejo Hospitalario Universitario de Albacete, Albacete, Spain

**Keywords:** ischemic stroke, transient ischemic attack (TIA), plasma, ischemic preconditioning (IPC), endogenous neuroprotection, biomarker (BM)

## Abstract

**Introduction:** Ischemic tolerance (IT) refers to a state where cells are resistant to the damaging effects caused by periods of ischemia. In a clinical scenario, the IT phenomenon would be activated by a recent transient ischemic attack (TIA) before an ischemic stroke (IS). The characterization of inflammatory protein expression patterns will contribute to improved understanding of IT.

**Methods:** A total of 477 IS patients from nine hospitals, recruited between January 2011 and January 2016, were included in the current study and divided in three groups: 438 (91.9%) patients without previous TIA (group 1), 22 (4.6%) patients who suffered TIA 24 h before IS (group 2), and 17 (3.5%) patients who suffered TIA between 24 h and 7 days prior to IS (group 3). An inflammatory biomarker panel (IL-6, NT-proBNP, hsCRP, hs-Troponin, NSE, and S-100b) on plasma and a cytokine antibody array was performed to achieve the preconditioning signature potentially induced by TIA phenomena. Primary outcome was modified rankin scale (mRs) score at 90 days.

**Results:** Recent previous TIA was associated with better clinical outcome at 90 days (median mRS of group 1: 2.0 [1.0–4.0]; group 2: 2.0 [0.0–3.0]; group 3: 1.0 [0–2.5]; *p* = 0.086) and smaller brain lesion (group 1: 3.7 [0.7–18.3]; group 2: 0.8 [0.3–8.9]; group 3: 0.6 [0.1–5.5] mL; *p* = 0.006). All inflammation biomarkers were down regulated in the groups of recent TIA prior to IS compared to those who did not suffer a TIA events. Moreover, a cytokine antibody array revealed 30 differentially expressed proteins between the three groups. Among them, HRG1-alpha (Fold change 74.4 between group 1 and 2; 74.2 between group 1 and 3) and MAC-1 (Fold change 0.05 between group 1 and 2; 0.06 between group 1 and 3) expression levels would better stratify patients with TIA 7 days before IS. These two proteins showed an earlier inflammation profile that was not detectable by the biomarker panel.

**Conclusion:** Inflammatory pathways were activated by transient ischemic attack, however the period of time between this event and a further ischemic stroke could be determined by a protein signature that would contribute to define the role of ischemic tolerance induced by TIA.

## Introduction

Ischemic tolerance (IT) refers to a state where cells are resistant to the damaging effects caused by periods of ischemia. IT was first identified in the heart (1), but it was subsequently found to also occur in the brain (2, 3). The IT mechanisms induced by brief periods of ischemia appear complex; it is an active process that triggers gene expression, metabolic signatures, and activation of signaling pathways (4, 5). The IT could be induced either by local, global and/or by remote ischemic preconditioning (IPC) (3, 6, 7). IPC consists of brief periods of ischemia that confers protection against later episodes of prolonged tissue ischemia (6). IPC phenomenon has been evidenced both *in vitro* and *in vivo* models (3, 8, 9). The IPC/IT paradigm allows study of neuroprotection and transient ischemic attacks (TIAs) are experimental strategies to study it. IT phenomenon is activated by TIA and it has a similar underlying mechanism as acute ischemic stroke (AIS) (10, 11).

In a clinical scenario, IT phenomenon is activated by a recent TIA before an AIS (12–16). An ischemic event is characterized by the occlusion of an arterial vessel that supplies blood to an area of the brain, resulting in a corresponding loss of neurological function. TIA consists in a brief episode of neurological dysfunction caused by ischemia, without structural damage, and it has the same underlying mechanism as AIS (17). Thus, patients with previous TIA before an AIS had better outcome than patients without previous TIA (12–16, 18). Moreover, a beneficial effect of TIAs on final infarct volumes has been demonstrated (12, 18). Similarly, ischemic presentations prior to acute myocardial infarction (AMI) are associated with lower short-term mortality after AMI, suggesting a natural ischemic preconditioning effect (19).

Inflammation plays a dual role in the ischemic stroke's pathophysiology: tissue repair and secondary brain injury effects. Inflammatory response is uniform in ischemic stroke, including activation of microglia and endothelial cells by oxidative stress and excitotoxicity, infiltration of peripheral leukocytes in affected areas and causing tissue damage (20, 21). Proinflammatory factors leads to upregulation of cell adhesion molecules on endothelial cells causing inflow of blood to the ischemic area and further activation of microglia and astrocytes. Cytokines are immunomodulating agents and they play a major role in cell activation, proliferation, and differentiation (22), they are barely detectable in the brain and they provoke and aggravate inflammatory response after stroke (23). Proinflammatory cytokines help to activate endothelial cells locally, but they are mainly released into circulation. Although the basic concept of inflammatory response after stroke is quite well-known, there is still missing information and the concept is constantly evolving.

Biomarkers are defined as cellular, biochemical or molecular alterations that are objectively measurable in biological samples such as human tissues, cells, or fluids; they are used as an indicator of a biological or clinical condition, often with potential diagnostic or prognostic value. Protein array is an emerging technology, it deems to be a versatile and robust tool to detect and/or quantify a large number of proteins present in a complex biological sample. Serum contains massive amounts of potentially pathophysiological information of ischemic stroke, so protein array of human plasma would provide a so-called signature which would significantly increase diagnostic/prognostic accuracy.

According to our understanding, the protein profile of the preconditioning signature induced by TIA phenomena has not been characterized. The first aim of the current study was to determine the inflammatory protein expression of an acute ischemic stroke patients cohort, all patients were assessed for an inflammation panel of six biomarkers [interleukin-6 (IL-6), N-terminal pro-B type natriuretic peptide (NT-proBNP), high-sensitivity C-reactive protein (hsCRP), high sensitive troponin (hsTroponin), neuron-specific enolase (NSE), S-100b protein]. Moreover, the second aim was to assess a biomarker discovery by a cytokine antibody array on a cohort of acute ischemic stroke patients. For that, the differential expression profile of 1,000 inflammation cytokines was performed by an antibody array-based technology on AIS patients with and without previous recent TIA phenomena.

## Materials and Methods

### Study Participants—Cohorts Description

A cohort of suspected acute ischemic stroke (AIS) patients (*n* = 756) was consecutively prospective recruited between January 2011 and January 2013 from the Hospital Universitari Arnau de Vilanova (HUAV) (Lleida, Spain), and between January 2014 and January 2016 from nine Hospitals in Spain (Hospital Universitari Arnau de Vilanova-Lleida, Hospital Universitario-Pamplona, Hospital del Mar-Barcelona, Hospital de son Espases-Mallorca, Hospital Parc Taulí-Sabadell, Hospital de Sant Pau-Barcelona, Hospital Ramón y Cajal-Madrid and Hospital de Albacete). The same recruitment protocol was followed at each site: TIA/AIS diagnose, neuroimaging confirmation and blood extraction. At Hospital admission, AIS patients were attended by a Neurologist within 24 h from symptoms onset and underwent neuroimaging diagnosis with at least cranial computed tomography. Patients with symptoms that persisted <24 h without clear evidence of acute ischemic lesion in the neuroimaging were excluded from the study (17) (tissue criteria). The other exclusion criteria were a modified Rankin Scale Score (mRS) > 3, duration of stroke symptoms more than 24 h and/or patients under 18 years old.

All blood samples were obtained within the first 24 h after the onset of symptoms acute ischemic stroke symptoms at the Hospital admission at the emergency room by standard venipuncture. Plasma, serum and buffy coat were obtained after centrifugation at 3,000 g at 4 °C for 10 min, and aliquoted into cryovials for immediate storage at −80 °C (Plataforma Biobancos PT17/0015/0027).

Patients were classified etiologically according to TOAST criteria (Trial of Org 10,172 in Acute Stroke Treatment) (24) as large-artery occlusive disease (LAA), small-vessel disease (SV), cardioembolic (CE), other cause (OC), or undetermined cause (UND). The following patient's characteristics were collected: age at admission, gender, vascular risk factors, prior treatments, revascularization therapies (recombinant tissue plasminogen activator: rt-PA; thrombectomy; rt-PA and thrombectomy; non-revascularization treatment), premorbid modified Rankin scale (mRS) and stroke severity by the National Institutes of Health Stroke Scale (NIHSS) at admission, 24 h and 7 days.

According to previous evidence (17, 25), patients were classified in three experimental groups defined by the time interval between recent TIA and ischemic stroke: (i) previous TIA within 24 h, (ii) previous TIA between 24 h and 7 days, and (iii) stroke without previous TIA.

Previous TIA was defined as a reversible episode of neurological deficit of ischemic origin that resolved completely within 24 h (26). Families of patients with language impairment or disturbances of consciousness and their general practitioners were interviewed to identify prior TIA events.

All baseline data was centrally monitored and queries were sent to the enrolling physicians of each center. The main clinical outcome was the degree of functional impairment at 3 months measured by mRS at 90 days. Patients were followed up by in-person interview in each recruiting Hospital. Moreover, final infarct volume was measured in 366 (76.7%) patients by diffusion-weighted magnetic resonance imaging (DWI) performed within 7 days (median 3.2 days: SD 1.8). One Neuroradiologist blinded to clinical features established the presence of DWI abnormalities. Furthermore, OsiriX v4.0 semi-automated segmentation tool and imaging software (Bernex, Switzerland) (27) was used to calculate the total volume of DWI abnormal signal intensity. Polygon tool was used to assess the presence of abnormalities, regions of interest (ROIs) were delineated between segmented slices and automatically interpolated, and the infarct volume determined (28).

This study protocol was approved by the Ethics Committee of the HUAV and all participating Hospitals (approval code: 928). Written informed consent was obtained from the patients or patients' surrogates. All procedures followed the guidelines of the Declaration of Helsinki developed by the World Medical Association (WMA) regarding ethical principles for medical research involving human subjects.

### Inflammatory Biomarkers Panel

IL-6, NT-proBNP, hsCRP, hs-Troponin, NSE, and S-100b levels were assessed in plasma samples (Hoffmann-La Roche, Basel, Switzerland) by an electrochemical chemiluminescence immunoassay using the COBAS 6000 e601 (Hoffmann-La Roche, Basel, Switzerland) at the Medical Laboratory of the HUAV. Three hundred microliter of plasma was used to perform the inflammatory biomarkers determination.

### Inflammation Antibody Array

To identify differentially expressed circulating proteins a cytokine antibody array was performed on eight groups of plasma pools. The cytokines were detected using RayBio® Biotin Label-based Antibody Array (L-Series, RayBiotech Life, Peachtree Corners, GA), a quantitative array platform using multiplexed sandwich ELISA-based technology, which detect 1,000 proteins.

**Figure 1:**
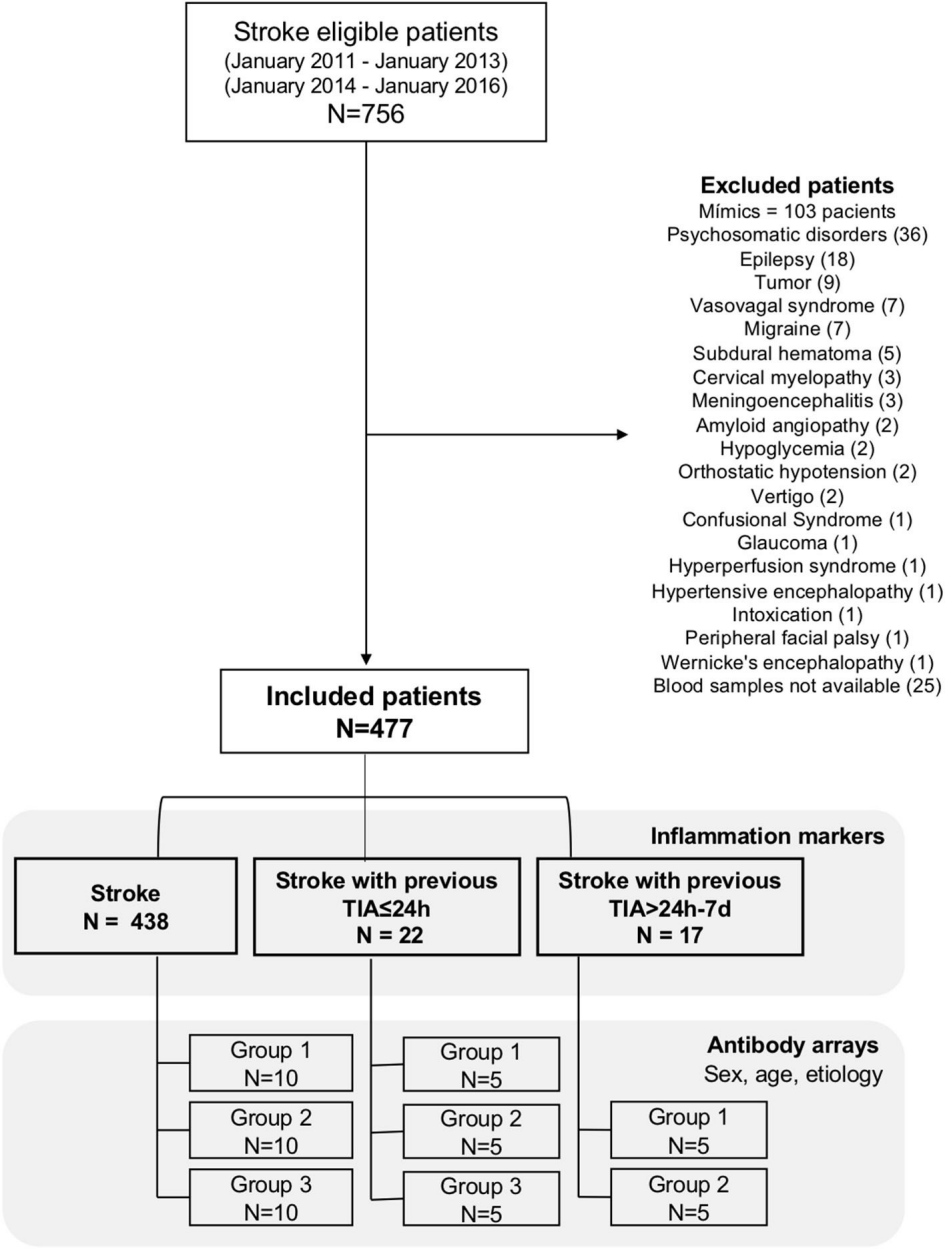
Flow diagram of recruited patients, excluded patients, and number of final included patients in inflammation markers and antibody arrays.

In the group of stroke patients without previous TIA, 30 patients were selected by sex, age, and etiology. Three subgroups were established with ten samples per subgroup and samples pooled within each subgroup. Same procedure was performed in the group of stroke with previous TIA 24 h before, where fifteen samples were pooled in three subgroups, and 10 samples were pooled in the group stroke with previous TIA between 24 h and 7 days in two subgroups (Figure 1). Briefly, 10 μl of each patient plasma sample were pooled in the corresponding subgroup. For each pooled subgroup, 30 μl were used according to the manufacturer's protocol. Finally, the signals were visualized by chemiluminescence (reading wave length 600PMT) and then imaged by GenePix Personal 4100A microarray scanner (Molecular Devices, San José, California, USA). The obtained pictures were analyzed with GenePix Pro Microarray Analysis (Molecular Devices, San José, California, USA) program.

### Data Analysis

Descriptive and frequency statistical analyses of demographic, etiological, and management variables; and inflammatory biomarkers panel were performed with SPSS for Mac, version 20. Categorical variables were shown as frequencies and percentages, and continuous variables as means and standard deviations. Inflammatory biomarkers were not normally distributed (P-P plot) and values were expressed as median (interquartile range).

First, we compared demographic, etiological, and management variables between the three groups attending the precedent of previous recent TIA. Statistical significance for intergroup differences was assessed by Pearson's chi-square test for categorical variables and the ANOVA *t-*test, Student's *t-*test, Kruskal-Wallis test or Mann-Whitney test for continuous variables. To calculate the sensitivity and specificity for biomarker cut-off values which allowed to discriminate AIS patients with previous recent TIA from AIS patients without previous TIA, a receiver operator characteristic (ROC) analysis was performed. Variables associated in the univariate analysis showing *p* < 0.10 were entered into a sequential logistic-regression model to identify variables independently associated with previous recent TIA (odds ratios, 95% confidence interval).

mRs at 90 days was the primary clinical endpoint. mRs ≤ 3 was considered as good outcome. We compared patients' characteristics between groups. In addition, simple logistic regression analysis was performed to identify variables associated with the occurrence of good outcome (odds ratios, 95% confidence interval).

Gene Ontology (GO) (29) and pathway analysis (PANTER: protein annotation through evolutionary relationship) (30) were conducted for proteins of interest using web-based tools (http://geneontology.org and http://www.pantherdb.org). Statistical significance was considered when *p* ≤ 0.05 and fold change (FC) criteria of FC > 2 or FC <0.5.

## Results

### Clinical Characterization of Experimental Groups/Cohort

Of 756 patients, 477 patients met the inclusion criteria (“stroke”), 22 (4.6%) patients had a previous TIA 24 h before the acute ischemic stroke (“stroke with previous TIA ≤ 24 h”) and 17 (3.5%) patients had a previous TIA between 24 h and 7 days before the acute ischemic stroke (“stroke with previous TIA 24 h−7 d”) (Figure 1).

The average age was 74.3 (±11.6) years and 253 (53.0%) patients were female. Patients included in the group stroke with previous TIA ≤ 24 h were significantly younger, they had more smoking habits, they were more under renin-angiotensin system blockers (Table 1). LAA subtype was significantly more frequent among patients with recent previous TIA than in patients without. In contrast, CE was the most frequent etiologic subtype in AIS without recent previous TIA.

**Table 1:** Clinical characteristics of all included patients by experimental groups.

	**All**	**Stroke (no previous TIA)**	**Stroke with previous TIA ≤ 24 h**	**Stroke with previous TIA 24 h−7 d**	**Comparison between all groups *p*-value^a^**	**Comparison between stroke vs. stroke with previous TIA ≤ 24 h *p*-value^a^**	**Comparison between stroke vs. stroke with TIA 24 h−7 d *p*-value^a^**
**Total** n	477	438 (91.8)	22 (4.6)	17 (3.5)			
**Age** mean ± SD years	74.3 ± 11.6	74.7 ± 11.4	67.82 ± 12.5	71.4 ± 11.3	0.013^*^	0.006^*^	0.232
**Baseline characteristics**							
Male sex	280 (53.0)	253 (57.8)	14 (63.6)	13 (76.5)	0.273	0.586	0.125
Alcoholism	34 (7.1)	32 (7.3)	2 (9.1)	0 (0.0)	0.502	0.675	0.618
Unknown	5 (1.0)	4 (0.9)		1 (5.9)			
Smoking habits	93 (19.5)	80 (18.3)	8 (36.36)	5 (29.41)	0.067	0.036^*^	0.252
Unknown	2 (0.4)	2 (0.5)					
Hypertension	357 (74.8)	332 (75.8)	14 (63.6)	11 (64.7)	0.260	0.190	0.289
Unknown	1 (0.2)	1 (0.2)					
Diabetes mellitus	129 (27.0)	120 (27.4)	5 (22.73)	4 (23.5)	0.835	0.622	0.717
Unknown	2 (0.4)	2 (0.5)					
Hyperlipidemia	209 (43.8)	192 (43.8)	12 (54.6)	5 (29.4)	0.292	0.324	0.239
Previous Atrial fibrillation	117 (24.5)	111 (25.3)	5 (22.7)	1 (5.9)	0.181	0.774	0.085
Unknown	2 (0.4)	2 (0.5)					
Ischemic heart disease	53 (11.1)	51 (11.6)	2 (9.1)	0 (0.0)	0.309	0.712	0.135
Unknown	1 (0.2)	1 (0.2)					
**Previous treatment**							
Antiplatelet treatment	177 (37.1)	167 (38.1)	4 (18.2)	6 (35.3)	0.163	0.071	0.808
Unknown	1 (0.2)	1 (0.2)					
Anticoagulation	60 (12.6)	56 (12.8)	3 (13.6)	1 (5.9)	0.692	0.933	0.387
Unknown	1 (0.2)	1 (0.2)					
Statins	167 (35.0)	154 (35.2)	10 (45.5)	3 (17.7)	0.191	0.329	0.194
Unknown	1 (0.2)	1 (0.2)					
Renin-angiotensin system blockers	153 (32.08)	142 (32.42)	6 (27.3)	5 (29.4)	0.899	0.030	0.788
Unknown	3 (0.63)	2 (0.46)	1 (4.55)				
**Management**					0.887	0.894	0.625
rt-PA administration	95 (19.9)	88 (20.1)	4 (18.2)	3 (17.7)			
rt-PA + Thrombectomy	33 (6.9)	32 (7.3)	1 (4.5)	0 (0.0)			
Thrombectomy alone	5 (1.0)	5 (1.1)	0 (0.0)	0 (0.0)			
**Stroke severity**							
NIHSS^†^
At admission	5 (3.0–11.0)	5 (3.0–12.0)	1 (0.0–4.3)	4 (0.5–5.5)	<0.001^*^	<0.001	0.033
24 h	3 (1.0–8.0)	4 (2.0–9.0)	1 (0.0–4.5)	2 (0.0–3.0)	0.002^*^	0.006	0.020
7 days	2 (0.0–5.0)	2 (0.0–5.0)	0.5 (0.0–3.5)	1 (0.0–2.0)	0.024^*^	0.061	0.039
mRs^‡^
At admission	0 (0.0–1.0)	0 (0.0–1.0)	0 (0.0–1.0)	0 (0.0–0.8)	0.531	0.264	0.930
7 days	2 (1.0–4.0)	2 (1.0–4.0)	1.5 (0.0–3.0)	2 (0.0–3.0)	0.030^*^	0.045	0.070
90 days	2 (1.0–3.0)	2 (1.0–4.0)	2 (0.0–3.0)	1 (0.0–2.5)	0.086	0.263	0.050
mRs 90 days ≤ 3	355 (75.9)	320 (90.1)	19 (86.4)	16 (94.1)	0.091	0.213	0.050
**Stroke Etiologies (TOAST)**					0.037^*^	0.085	0.055
LAA	77 (16.1)	65 (14.8)	7 (31.8)	5 (29.4)			
CE	203 (42.6)	194 (44.3)	6 (27.3)	3 (17.7)			
Lacunar	72 (15.1)	62 (14.2)	5 (22.7)	5 (29.4)			
Undeterminate	115 (24.1)	109 (24.9)	3 (13.6)	3 (17.7)			
Other causes	10 (2.1)	8 (1.8)	1 (4.6)	1 (5.9)			
**DWI vol. Median (IQR) mL**	2.8 (0.6–17.7)	3.7 (0.8–18.3)	0.8 (0.3–2.9)	0.6 (0.15.5)	0.006^*^	0.044	0.011

*Plus–minus values are means ± SD. Percentages are shown in parentheses as appropriate. Percentages may not total 100 because of rounding*.

† *Scores on the National Institutes of Health Stroke Scale range from 0 to 42, with higher score indicating impairment after stroke. Values are presented as median and interquartile range*.

‡ *Scores on modified Rankin Score range from 0 to 5, with higher scores indicating major disability or dependence in the daily activities. Values are presented as median and interquartile range*.

a *p-values correspond to the chi-square test for trend for ordered qualitative variables and the Pearson's. chi-square test for non-ordered qualitative variables and have been obtained excluding the patients with missing values*.

* *p ≤ 0.05*.

*TIA, Transient ischemic attack; rt-PA, recombinant tissue plasminogen activator; mRS, modified Rankin Score; NIHSS, National Institutes of Health Stroke Scale; LAA, large-artery occlusive disease; CE, cardioembolic*.

Clinical characteristics of patients with previous TIA showed a significant less severe stroke at admission by NIHSS's score. Infarct volumes of stroke with previous TIA ≤ 24 h group and stroke with previous TIA 24 h−7 d group were significantly reduced. Furthermore, outcome at 7 and 90 days measured by disability or dependence in daily activities (mRS) was better in previous TIA ≤ 7 d patients than non-previous TIA patients (Table 1), these patients had better significant outcomes at 7 days and a tendency at 90 days as well (*p* = 0.086), measured by NIHSS and mRs score. Table 2 shows variables associated with mRS at 90 days < =3. In addition to previous recent TIA (Odds ratio 2.98, 95% CI 1.04–8.58) sex male and small vessel disease etiology were related to better outcomes. In contrast, age, basal NIHSS, previous atrial fibrillation and cardioembolic etiology were associated to mRS > 3.

**Table 2:** Univariate analysis and simple logistic regression analysis of variables associated with outcomes at follow-up (90 days).

	**All**	**mRS > 3**	**mRS ≤ 3**	***p*-value^a^**	**OR (95%CI)**	***p*-value**
**Total** n	468	175 (37.4)	293 (62.6)	–	–	–
**Age** mean ± SD years	74.3 ± 11.5	80.6 ± 9.3	72.3 ± 11.5	<0.001	0.92 (0.89–0.94)	<0.001^*^
**Baseline characteristics**						
Male sex	276 (59.9)	49 (43.4)	226 (63.7)	<0.001	2.29 (1.49–3.52)	<0.001^*^
Hypertension	351 (75.2)	91 (80.5)	260 (73.4)	0.129	0.67 (0.40–1.13)	0.131
Unknown	1 (0.2)					
Diabetes mellitus	127 (27.3)	28 (25.0)	99 (28.0)	0.539	1.17 (0.72–1.90)	0.539
Unknown	2 (0.4)					
Hyperlipidemia	203 (43.4)	44 (38.9)	158 (44.5)	0.298	1.26 (0.82–1.94)	0.298
Previous Atrial fibrillation	114 (24.4)	37 (32.7)	77 (21.8)	0.018	0.57 (0.36–0.91)	0.019^*^
Unknown	1 (0.2)					
Ischemic heart disease	52 (11.1)	12 (10.6)	40 (11.3)	0.849	1.07 (0.54–2.12)	0.849
Unknown	1 (0.2)					
Stroke with previous TIA ≤ 7 days	39 (8.3)	4 (3.5)	35 (9.9)	0.034	2.98 (1.04–8.58)	0.043^*^
**Management**						
rt-PA administration	93 (19.9)	29 (25.7)	64 (18.0)	0.368	0.89 (0.65–1.22)	0.464
rt-PA + Thrombectomy	32 (6.8)	7 (6.2)	25 (7.0)			
Thrombectomy alone	5 (1.1)	1 (0.9)	4 (1.1)			
**Stroke severity**						
NIHSS at admission^†^	5.0 (3.0–11.0)	15.0 (9.0–20.0)	4.0 (2.0–8.0)	<0.001	0.82 (0.79–0.86)	<0.001^*^
Basal mRS^‡^	0.0 (0.0–1.0)	0.0 (0.0–1.0)	0.0 (0.0–0.0)	<0.001	0.55 (0.43–0.71)	<0.001^*^
**Stroke Etiologies (TOAST)**						
LAA	77 (16.5)	17 (15.0)	60 (16.9)	0.643	1.15 (0.64–2.06)	0.643
CE	200 (42.7)	63 (55.8)	138 (38.9)	0.002	0.51 (0.33–0.77)	0.002^*^
Small vessel	70 (15.0)	4 (3.5)	66 (18.6)	<0.001	6.22 (2.22–17.48)	0.001^*^
Undeterminate	111 (23.7)	27 (23.9)	83 (23.4)	0.911	0.97 (0.59–1.60)	0.911
Other causes	10 (2.1)	2 (1.8)	8 (2.3)	0.757	1.28 (0.27–6.11)	0.757
**DWI vol. Median (IQR) mL**	2.8 (0.6–17.7)	38.6 (10.4–142.4)	1.6 (0.5–9.3)	<0.001	0.97 (0.96–0.98)	<0.001^*^

*Plus–minus values are means ± SD. Percentages are shown in parentheses as appropriate. Percentages may not total 100 because of rounding*.

† *scores on the National Institutes of Health Stroke Scale range from 0 to 42, with higher score indicating impairment after stroke. Values are presented as median and interquartile range*.

‡ *Scores on modified Rankin Score range from 0 to 5, with higher scores indicating major disability or dependence in the daily activities. Values are presented as median and interquartile range*.

a *p-values correspond to the chi-square test for trend for ordered qualitative variables and the Pearson's. chi-square test for non-ordered qualitative variables and have been obtained excluding the patients with missing values*.

* *p ≤ 0.05*.

*TIA, Transient ischemic attack; rt-PA, recombinant tissue plasminogen activator; mRS, modified Rankin Score; NIHSS, National Institutes of Health Stroke Scale; LAA, large-artery occlusive disease; CE, cardioembolic*.

### Inflammatory Biomarkers Panel

**Figure 2:**
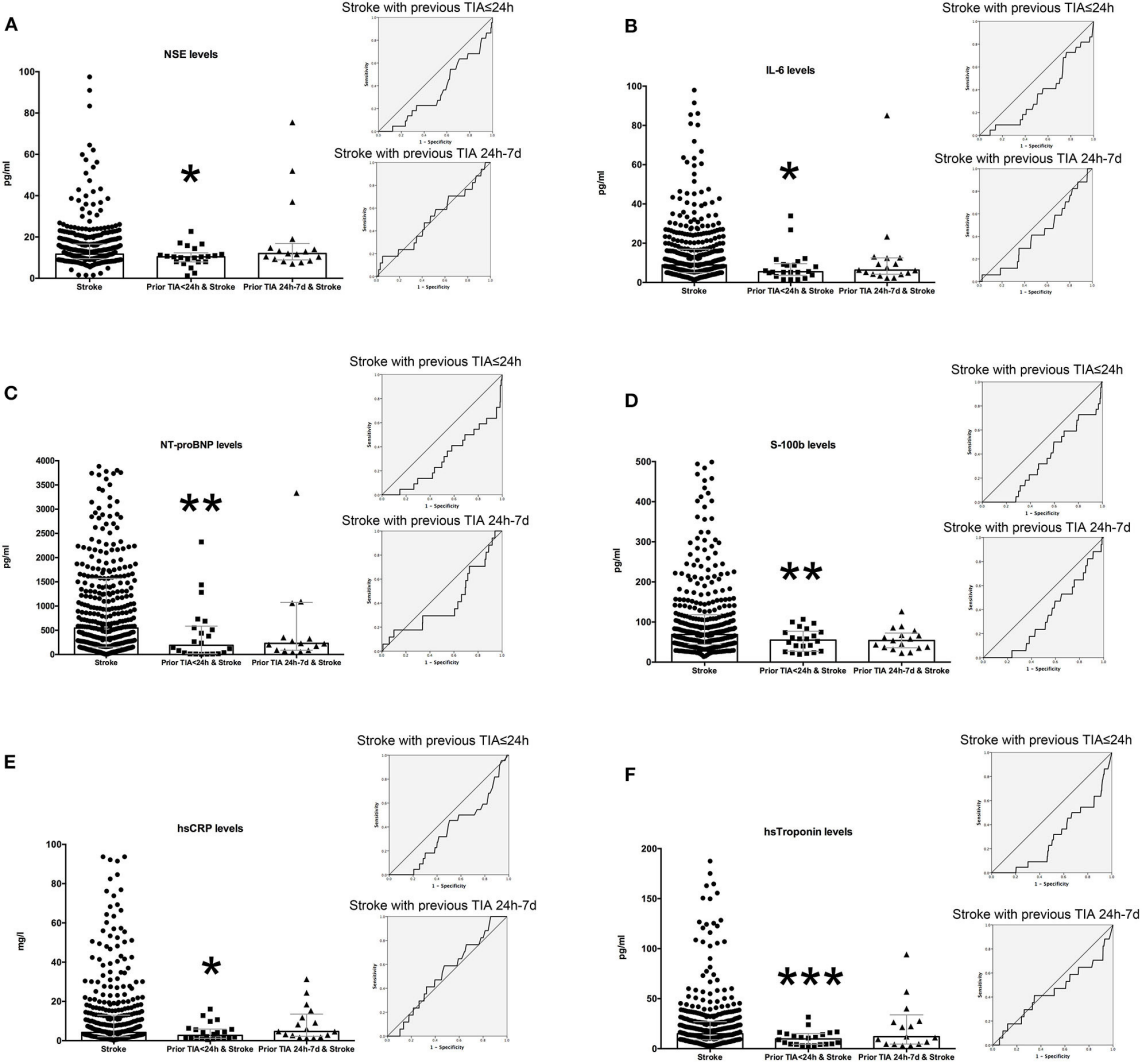
Plasma levels of inflammatory markers according to preconditioning induced by TIA. The levels of NSE **(A)**, IL-6 **(B)**, NT-proBNP **(C)**, S-100b **(D)**, hsCRP **(E)**, and hsTroponin **(F)** were determined by chemiluminescence immunoassay. Differences in levels of inflammatory markers were analyzed using non-parametric test with *p* < 0.05 being significant. Graphs represent median with interquartile range (gray color). Receiver operating characteristic (ROC) curve for stroke with previous TIA ≤ 24 h and stroke with previous TIA (24 h−7 d) were an insert for each biomarker. Area under the curve (AUC) for NSE is 0.359, *p* = 0.026 for previous TIA ≤ 24 h; and 0.507, *p* = 0.921 for previous TIA 24 h−7 d. AUC for IL6 is 0.358, *p* = 0.024 for previous TIA ≤ 24 h; and 0.415, *p* = 0.236 for previous TIA 24 h−7 d. AUC for NT proBNP is 0.303, *p* = 0.002 for previous TIA ≤ 24 h; and 0.408, *p* = 0.200 for previous TIA 24 h−7 d. AUC for S100B is 0.344, *p* = 0.014 for previous TIA ≤ 24 h; and 0.342, *p* = 0.027 for previous TIA 24 h−7 d. AUC for hsCRP is 0.374, *p* = 0.046 for previous TIA ≤ 24 h; and 0.529, *p* = 0.689 for previous TIA 24 h−7 d. AUC for hs troponin is 0.298, *p* = 0.001 for previous TIA ≤ 24 h; and 0.443, *p* = 0.427 for previous TIA 24 h−7 d. *p-value between 0.025 and 0.05; **p-value between 0.01 and 0.025; ***p < 0.01.

NSE, IL-6, NT-proBNP, S-100b, hsCRP levels, and hsTroponin showed significantly lower circulating levels in stroke patients with previous TIA ≤ 24 h (Figure 2). Different cut-off values were obtained for the biomarkers on different endpoints: TIA ≤ 24 h and TIA between 24 h and 7 days. IL-6 <5.1 pg/ml has a sensitivity of 68% and a specificity of 27% for TIA ≤ 24 h and a sensitivity of 65% and a specificity of 29% for TIA between 24 h and 7 days. NT-proBNP <82.25 pg/ml has a sensitivity of 64% and a specificity of 13% for TIA ≤ 24 h. NT-proBNP <203.6 pg/ml has a sensitivity of 65% and a specificity of 28% for TIA between 24 h and 7 days. S100b <41.4 has a sensitivity of 64% and a specificity of 21% for TIA ≤ 24 h. S100b <43.2 pg/ml with a sensitivity of 65% and a specificity of 24% for TIA between 24 h and 7 days. HsTroponin <6.05 pg/ml has a sensitivity of 64% and a specificity of 15% for TIA ≤ 24 h. HsTroponin <9.35 pg/ml has a sensitivity of 65% and a specificity of 28% for TIA between 24 h and 7 days.

Table 3 shows the binary regression model to assess the relation of recent TIA prior to acute ischemic stroke. Unfortunately, none of the assessed biomarkers showed a statistically significant prediction of previous recent TIA.

**Table 3:** Binary regression model for pre-conditioning effect of TIA on acute ischemic patients.

	**Stroke with previous TIA** **≤** **24 h**		**Stroke with previous TIA 24 h−7 d**	
**Variables**	**OR (95% CI)**	***p*-value^a^**	**OR (95% CI)**	***p*-value^a^**
Age	0.98 (0.94–1.02)	0.358	0.98 (0.94–1.03)	0.458
LAA	2.27 (0.87–5.96)	0.096	2.36 (0.79–7.05)	0.125
IL-6 <5.1 pg/mL	0.65 (0.22–1.89)	0.429	1.22 (0.38–3.93)	0.744
NT-ProBNP <82.25 pg/mL	1.90 (0.57–6.38)	0.300	–	–
NT-ProBNP <203.6 pg/mL	–	–	0.86 (0.25–2.99)	0.808
S100b <41.4 pg/mL	1.87 (0.59–5.91)	0.288	–	–
S100b <43.15 pg/mL	–	–	1.40 (0.47–4.15)	0.548
hsTroponin <6.05 pg/mL	1.87 (0.59–5.91)	0.288	–	–
hsTroponin <9.35 pg/mL	–	–	1.03 (0.29–3.65)	0.960

a *p ≤ 0.05*.

*OR, Odds ratio; TIA, Transient ischemic attack; LAA, large-artery atherosclerosis; IL-6, interleukin-6; NT-proBNP, N-terminal pro-B type natriuretic peptide; hsTroponin, high sensitive troponin*.

### Inflammation Antibody Array

A cytokine antibody array was performed in three groups of pooled patients to identify differentially expressed cytokine circulating proteins. Group 1 contained 30 ischemic stroke (IS) patients without previous TIA divided in three subgroups (10 samples each); group 2 contained 15 patients with TIA 24 h prior to IS divided in three subgroups (5 samples each), and group 3 had 10 patients with TIA between 24 h and 7 days prior IS divided in two subgroups (5 samples each). Each group/subgroup of pooled samples were balanced by sex, age, and etiology (Table 4).

**Table 4:** Clinical characteristics of included patients in the inflammation array experiment.

	**Stroke (no previous TIA) (*N =* 30)**				**Stroke and previous TIA < 24 h (*N =* 15)**				**Stroke and previous TIA 24 h−7 d (*N =* 10)**			**Intergroup differences**
	**Group 1**	**Group 2**	**Group 3**	***p*-value^a^**	**Group 1**	**Group 2**	**Group 3**	***p-*value^a^**	**Group 1**	**Group 2**	***p*-value^a^**	***p*-value^a^**
Total n	10	10	10		5	5	5		5	5		
Age mean ± SD years	70.0 ± 8.8	70.4 ± 7.5	67.5 ± 11.3	0.756	66.8 ± 16.4	66.6 ± 6.5	67.6 ± 9.4	0.990	70.0 ± 12.2	70.0 ± 16.3	1	0.704
**Baseline characteristics**												
Sex, male (%)	8 (80.0)	9 (90.0)	8 (80.0)	0.787	4 (80.0)	4 (80.0)	4 (80.0)	1	4 (80.0)	4 (80.0)	1	0.946
Alcoholism	1 (10.0)	3 (30.0)	2 (20.0)	0.535	0 (0.0)	1 (20.0)	0 (0.0)	0.343	0 (0.0)	0 (0.0)	–	0.124
Smoking habit	2 (20.0)	2 (20.0)	5 (50.0)	0.276	1 (20.0)	2 (40.0)	2 (40.0)	0.741	2 (40.0)	1 (20.0)	0.114	0.921
Hypertension	9 (90.0)	8 (80.0)	8 (80.0)	0.787	3 (60.0)	2 (40.0)	4 (80.0)	0.435	3 (60.0)	4 (80.0)	0.490	0.198
Diabetes Mellitus	3 (30.0)	4 (40.0)	6 (60.0)	0.387	1 (20.0)	1 (20.0)	1 (20.0)	1	0 (0.0)	2 (40.0)	0.114	0.241
Hyperlipidemia	8 (80.0)	5 (50.0)	6 (60.0)	0.366	2 (40.0)	3 (60.0)	4 (80.0)	0.435	3 (60.0)	2 (40.0)	0.537	0.315
Previous Atrial fibrillation	2 (20.0)	2 (20.0)	1 (10.0)	0.787	1 (20.0)	0 (0.0)	1 (20.0)	0.562	1 (20.0)	0 (0.0)	0.292	0.649
Ischemic heart disease	1 (10.0)	3 (30.0)	4 (40.0)	0.303	1 (20.0)	0 (0.0)	1 (20.0)	0.562	0 (0.0)	0 (0.0)	–	0.071
**Previous treatment**												
Antiplatelet	2 (20.0)	4 (40.0)	3 (30.0)	0.621	3 (60.0)	0 (0.0)	1 (20.00)	0.092	0 (0.0)	2 (40.0)	0.114	0.707
Anticoagulation	1 (10.0)	0 (0.0)	1 (10.0)	0.585	0 (0.0)	0 (0.0)	0 (0.00)	–	1 (20.00)	0 (0.00)	0.292	0.591
Statins	6 (60.0)	4 (40.0)	4 (40.0)	0.585	2 (40.0)	3 (60.0)	3 (60.00)	0.765	1 (20.00)	2 (40.00)	0.490	0.295
Renin-angiotensin system blockers	3 (30.00)	2 (20.00)	3 (30.00)	0.843	2 (40.00)	0 (0.00)	3 (60.00)	0.170	1 (20.00)	1 (20.00)	1	0.634
**Management**												
rt-PA	0 (0.0)	2 (20.0)	0 (0.0)	0.307	0 (0.0)	1 (20.0)	1 (20.0)	0.552	0 (0.0)	0 (0.0)	1	0.500
rt-PA + Thrombectomy	2 (20.0)	2 (20.0)	1 (10.0)		0 (0.0)	1 (20.0)	0 (0.0)		0 (0.0)	0 (0.0)		
Thrombectomy	0 (0.00)	0 (0.0)	1 (10.0)		0 (0.0)	0 (0.0)	0 (0.0)		0 (0.0)	0 (0.0)		
**Stroke severity and outcome**												
NIHSS Basal^†^	4.5 (3.0–9.8)	4 (0.0–11.8)	4 (2.8–6.5)	0.644	0 (0.0–6.0)	1 (0.5–11.5)	3 (0.0–6.5)	0.688	0 (0.0–2.5)	3 (0.0–9.0)	0.421	0.086
NIHSS 24 h	3.5 (1.8–7.3)	2 (0.0–4.0)	4.5 (2.5–8.0)	0.268	0 (0.0–2.5)	0.5 (0.0–6.3)	2 (0.0–4.5)	0.767	0 (0.0–1.5)	1 (0.0–11.0)	0.421	0.016^*^
NIHSS 7 days	2.5 (1.00–7.25)	1.5 (0.0–3.0)	3 (1.0–5.0)	0.212	0 (0.0–2.5)	1 (0.0–4.5)	0 (0.0–3.5)	0.865	0 (0.00–1.5)	0 (0.00–9.5)	1	0.059
mRs basal^‡^	0 (0.0–1.3)	0 (0.0–0.5)	0 (0.0–0.3)	0.819	0 (0.0–0.0)	0 (0.0–0.0)	0 (0.0–0.0)	0.472	0 (0.0–1.5)	0.5 (0.0–1.8)	0.343	0.790
mRS 7 days	3 (1.0–4.0)	1 (1.0–3.0)	2 (1.0–4.0)	0.258	0 (0.0–2.5)	1 (0.0–2.50)	0 (0.0–3.0)	0.939	0 (0.0–2.0)	0 (0.0–4.0)	0.690	0.018
mRS 90 days	3 (0.8–4.0)	1 (0.0–1.5)	1 (0.75–3.25)	0.259	0 (0.0–2.0)	0 (0.0–2.5)	2 (0.0–3.0)	0.578	1 (0.0–2.0)	0 (0.00–2.5)	0.841	0.160
**Stroke Etiologies (TOAST)**												
LAA	4 (40.00)	5 (50.00)	3 (30.00)	0.959	2 (40.00)	2 (40.00)	2 (40.00)	1	2 (40.00)	1 (20.00)	0.881	0.999
CE	2 (20.00)	2 (20.00)	2 (20.00)		1 (20.00)	1 (20.00)	1 (20.00)		1 (20.00)	1 (20.00)		
Lacunar	2 (20.00)	2 (20.00)	2 (20.00)		1 (20.00)	1 (20.00)	1 (20.00)		1 (20.00)	2 (40.00)		
Indeterminate	2 (20.00)	1 (10.00)	3 (30.00)		1 (20.00)	1 (20.00)	1 (20.00)		1 (20.00)	1 (20.00)		
DWI vol. Median (IQR) mL	3.3 (0.3–41.3)	0.6 (0.3–57.5)	1.7 (0.5–4.4)	0.963	1.3 (0.3–2.7)	0.8 (0.2–6.5)	0.5 (0.1–7.3)	0.564	0.8 (0.1–14.4)	0.1 (0.1–68.7)	0.548	0.083

*Plus–minus values are means ± SD. Percentages are shown in parentheses as appropriate*.

a *p-values correspond to the chi-square test for trend for ordered qualitative variables and the Pearson's. chi-square test for non-ordered qualitative variables and have been obtained excluding the patients with missing values*.

* *p ≤ 0.05*.

*TIA, Transient ischemic attack; rt-PA, recombinant tissue plasminogen activator; mRS, modified Rankin Score; NIHSS, National Institutes of Health Stroke Scale; LAA, large-artery occlusive disease; CE, cardioembolic; DWI, diffusion weighted imaging*.

† *Scores on the National Institutes of Health Stroke Scale range from 0 to 42, with higher score indicating impairment after stroke. Values are presented as median and interquartile range*.

‡ *Scores on modified Rankin Score range from 0 to 5, with higher scores indicating major disability or dependence in the daily activities. Values are presented as median and interquartile range*.

A total of 27 proteins exhibit significantly different expression between stroke and stroke with previous TIA ≤ 24 h (Table 5). Of these, 26 proteins showed lower expression levels in patients with TIA ≤ 24 h prior to ischemic stroke. Only HRG1-alpha showed higher expression levels in this group (fold change = 74.474).

**Table 5:** Differentially expressed proteins in acute ischemic stroke patients (no previous TIA) and stroke patients with previous TIA within 24 h.

**Protein (relative expression)**	**Stroke (no previous TIA)**	**Stroke with previous TIA ≤ 24 h**	***p*-value^a^**	**Fold-change^b^**	**Regulation (TIA ≤ 24 h vs. stroke)**
ACTIVIN B	144.054	23.629	0.042	0.164	Down
ANGIOPOIETIN-4	178.218	12.983	0.013	0.073	Down
ALK-6	7519.000	384.996	0.041	0.051	Down
CCR9	10713.000	856.717	0.032	0.080	Down
CRIM 1	134.054	12.783	0.007	0.095	Down
FGF R4	6885.000	1314.000	0.032	0.191	Down
WFIKKNRP	211.300	38.228	0.032	0.181	Down
HRG1-alpha	93.684	6977.000	0.034	74.474	Up
IFN-alpha	108.243	10.848	0.001	0.100	Down
IGFBP-7	157.790	3.920	0.008	0.025	Down
IL-1 F10	124.106	17.655	0.012	0.142	Down
IL-17	116.802	19.712	0.015	0.169	Down
IL-3	7520.000	69.126	0.008	0.009	Down
IL-6 R	235.073	77.546	0.019	0.330	Down
LEPTIN (OB)	10853.000	291.024	0.018	0.027	Down
LIPOCALIN-2	10108.000	478.070	0.041	0.047	Down
MAC-1	6955.000	378.802	0.004	0.054	Down
MMP-12	136.681	21.665	0.003	0.159	Down
MMP-15	169.020	10.369	0.014	0.061	Down
MMP-19	149.643	14.739	0.022	0.098	Down
GPNMB	2670.000	91.088	0.022	0.034	Down
CCL25	124.901	4.834	0.004	0.039	Down
TIMP-1	255.223	6.519	0.005	0.026	Down
TNFRSF1B	87.092	7.174	0.028	0.082	Down
TNFSF10	82.890	3.259	0.026	0.039	Down
TNFRSF10D	86.505	9.687	0.007	0.112	Down
VEGF R2 (KDR)	121.142	4.217	0.013	0.035	Down

a *Mann-Whitney U-test and Bonferroni adjustment. Statistical significance was considered when p-value <0.05*.

b *Fold-change analysis. Statistical significance was considered when FC > 2 or FC <0.5*.

*ALK-6, Activin-like Kinase; BMPR-IB, bone morphogenetic protein receptor IB; CCR9, C-C motif chemokine receptor 9; CRIM 1, cysteine rich transmembrane BMP regulator 1; FGF R4, fibroblast growth factor receptor 4; GASP-1, G protein-coupled receptor associated sorting protein 1; GPNMB, Glycoprotein non-metastatic melanoma B; HRG-alpha, histidine-rich glycoprotein alpha; IGFBP, insulin like growth factor binding protein; IL, inteleukin; IFN-alpha, interferon-alpha; MAC-1, macrophage-1 antigen; MMP, metalloproteinase; TECK, thymus-expressed chemokine; TIMP-1, tissue inhibitor of matrix metalloproteinase-1; TNFRSF1B, TNF receptor superfamily member 1B; TNFSF10, TNF superfamily member 10; TRAIL, TNF-related apoptosis-inducing ligand; TIA, Transient ischemic attack; TNF, tumor necrosis factor; VEGF, vascular endothelial growth factor; WFIKKNRP, WAP, Follistatin/kazal, Immunoglobulin, Kunitz, and Netrin domain-containing 2*.

**Figure 3:**
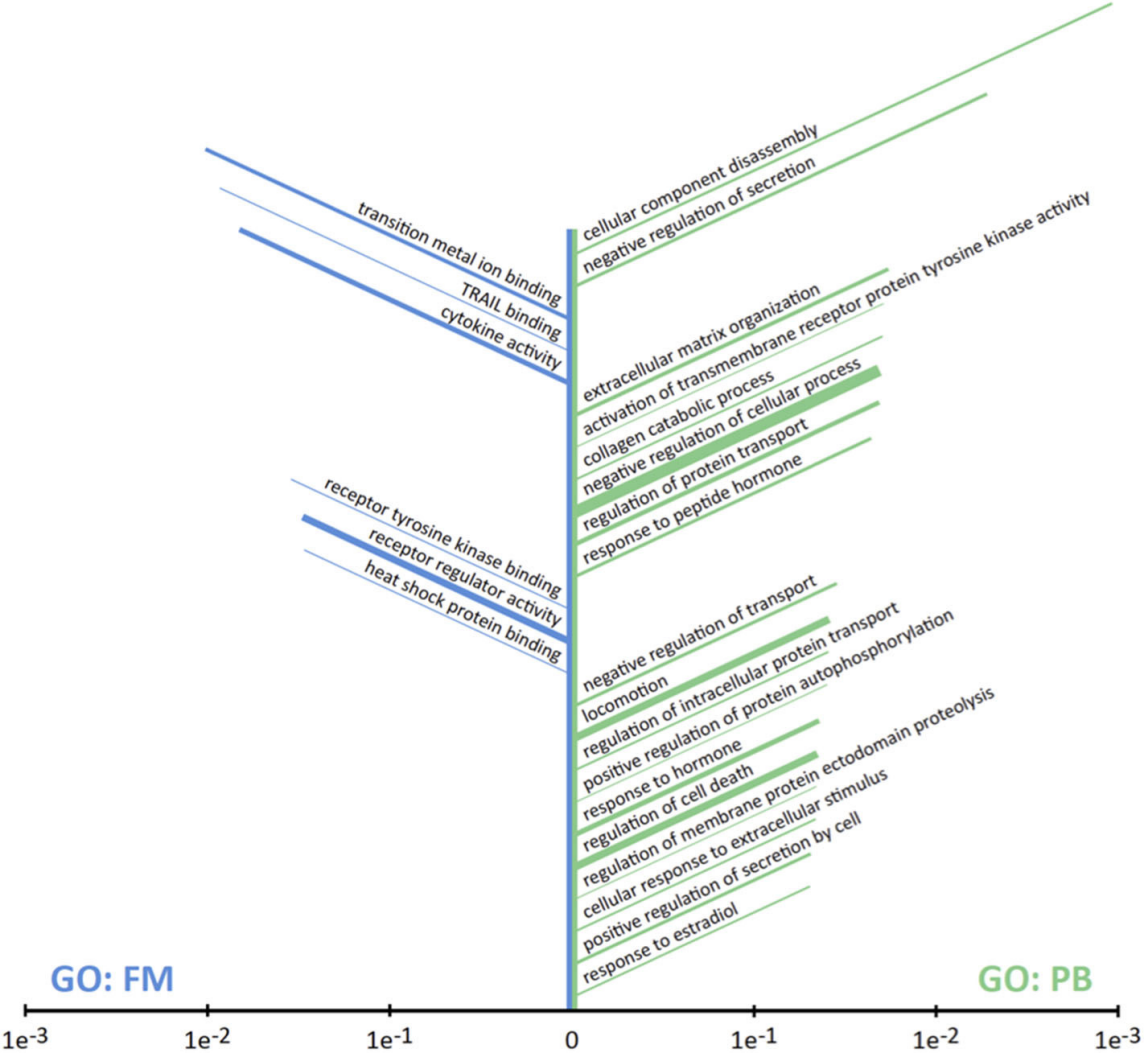
Results of GO Enrichment analysis on the set of 30 differentially expressed proteins. Terms are sorted according to *P*-value with the most significantly enriched terms at the top: GO terms for molecular functions (GO:FM, left, blue); GO terms for biological processes (GO:PB, right, green). The length of each bar correlates with *P*-value while the width indicates the number of proteins in the set associated with the term. Those functions or process that only one protein was involved were discarded.

The comparison of stroke patients without previous TIA and stroke patients with previous TIA between 24 h and 7 days before ischemic stroke showed five statically significant proteins: AMPHIREGULIN, HRG1-alpha, MAC-1, ONCOSTATIN M, UBIQUITIN+1 (Table 6). Three of them were down-regulated (AMPHIREGULIN, MAC-1, and ONCOSTATIN M) and two up-regulated (HRG1-alpha and UBIQUITIN+1). Interestingly, MAC-1 and HRG1-alpha showed a different relative expression level on both comparisons by prior TIA. The presence of a previous TIA showed decreased levels of MAC-1 and increased levels of HRG1-alpha in both time-windows (≤ 24 h and 24 h−7 d). Finally, PANTHER was used to perform gene ontology (GO) analysis on the inflammation antibody array's obtained results, the significantly enriched molecular function was the transition metal ion binding and the major number of proteins were associated with cytokine activity and receptor regulator activity. The enriched biological processes were cellular component disassembly and negative regulation of secretion; most of the enriched proteins were related with negative regulation of cellular processes, locomotion and regulation of cell death (Figure 3).

**Table 6:** Differentially expressed proteins on acute ischemic stroke patients (no previous TIA) and stroke patients with previous TIA between 24 h and 7 days.

**Protein (relative expression)**	**Stroke (no previous TIA)**	**Stroke with previous TIA 24 h−7 d**	***p*-value^a^**	**Fold-change^b^**	**Regulation (TIA 24 h−7 d vs. stroke)**
AMPHIREGULIN	149.731	25.127	0.002	0.168	Down
HRG1 ALPHA	93.684	6955.000	0.049	74.239	Up
MAC-1	6955.000	427.177	0.025	0.061	Down
ONCOSTATIN M	4116.000	897.950	0.029	0.218	Down
UBIQUITIN+1	182.943	3592.000	0.001	19.635	Up

a *Mann-Whitney U-test and Bonferroni adjustment. Statistical significance was considered when p-value <0.05*.

b *Fold-change analysis. Statistical significance was considered when FC > 2 or FC <0.5*.

*TIA, Transient ischemic attack; MAC-1, macrophage-1 antigen; HRG-alpha, heuregulin 1-alpha*.

## Discussion

In our study we observed a clinical translation of the preconditioning phenomena induced by an episode of TIA before acute ischemic stroke (AIS). Recent previous TIA was associated with better clinical outcome and smaller brain lesion. Interestingly, we observed significant low levels of inflammation-related biomarkers and using a cytokine array technology we describe up to 30 proteins, which would be potential inflammatory biomarkers with differential expression among ischemic stroke patients with previous recent TIA. Only two of them were upregulated: HRG1-alpha and Ubiquitin+1. Interestingly, MAC-1, and HRG1-alpha were significantly expressed in both groups of TIA patients (TIA ≤ 24 h and TIA 24 h−7 d) and they might be potential biomarkers to stratify patients with previous TIA events.

Prior TIA induces IT and it seems to activate an additional adaptive response based on inflammation biomarkers. The role of inflammation in the ischemic cascade after AIS is well-known. Following ischemic stroke, microglia and astrocytes are activated within hours, leading to the production of cytokines and chemokines and resulting in infiltration of leukocytes (31, 32). During the acute phase of stroke, lasting from minutes to hours, the injured tissue releases reactive oxygen species (ROS) and proinflammatory factors, such as chemokines and cytokines, which induce the expression of adhesion molecules on leukocytes and on cerebral endothelial cells that in turn promote adherence and trans-endothelial transfer of leukocytes (33). It has been described how preconditioning, among other actions, induces the capacity of the brain for self-protection by suppressing inflammation and dampening post-ischemic microglial activation (6). In addition, the inflammation effect plays a role in remote ischemic conditioning mediating neuroprotection against stroke (7).

IL-6 is an inflammatory cytokine upregulated by cerebral ischemia (34), but it has also been described previously how IL-6 levels among AIS patients with previous recent TIA are lower than AIS patients without prior TIA (12). A recent meta-analysis of 24 studies confirmed an unlikely translation of IL-6 into clinical practice for the prognosis of AIS (35); however, drugs that decreases plasma levels of IL-6 like anakinra (a recombinant IL-1 receptor antagonist) reduces infarct volume in experimental models (36). Our results showed a similar expression pattern, specifically a reduction of IL-6 levels in the group of ischemic stroke with previous TIA up to 24 h.

S100b levels were decreased in both groups with prior TIA before ischemic stroke. S100b could be found in both CSF and blood serum of different brain diseases like brain tumors, neuroinflammatory and neurodegenerative disorders, psychiatric disorders, cerebral infections, subarachnoid hemorrhage, acute brain injury, and cerebral infarction (37, 38). It is detected in glial cells of the central nervous system such as oligodendrocytes, ependymocytes, astrocytes, neuronal progenitor cells, and others (12). In the brain ischemia cellular microenvironment, S100b is released as a molecule that triggers an inflammatory response (39). For that, a reduction on S100b levels would be related with a reduction on the inflammatory reaction.

Lower levels of hsTroponin and NT-ProBNP were observed in patients with previous recent TIA. Elevated levels of both biomarkers are associated with unfavorable course of stroke and etiological cardioembolic type (40–43). Recently, the American Heart Association recommended to obtain a baseline troponin level in all patients with AIS, because stroke-associated cardiac dysfunction was detected by elevated troponin levels and associated with unfavorable short- and long-term outcomes after AIS (44).

Our results showed that previous TIA was more frequent among large artery atherosclerosis (LAA) etiology, as described previously (12, 14, 45). The overrepresentation of LAA etiology type and the smaller infarction size could interfere in our obtained results. In the multivariate analysis, no biomarker emerged as an independent predictor of previous recent TIA, although it is well-known the role of inflammation in the pathogenesis of atherosclerosis (46). This limitation was overcome by adjusting the groups of the inflammation antibody array by etiology and other variables, age and sex.

Cytokines are immunomodulating agents and they play a major role in cell activation, proliferation, and differentiation (22). Cytokines are generally small pleiotropic polypeptides (8–26 kDa) barely detectable in the brain with their receptors constitutively expressed at very low levels. Cytokines play a major role upregulating the expression of cell adhesion molecules (CAM) (47, 48). IL-6, interleukin 1β (IL-1β) and tumor necrosis factor-alpha (TNF-α) are the major proinflammatory cytokines that provoke and aggravate an inflammatory response after stroke (23, 49). Matrix metalloproteinases (MMPs) which are a large family of proteolytic enzymes that degrade all components of extracellular matrix were also identified (50). Although, MMPs act as proinflammatory factor, they are also important for normal physiological function such as neuronal regeneration, cell proliferation, angiogenesis, and apoptosis (51). In addition, in our study previous TIA was related to cytokines downregulation implicated in molecular functions and biological processes previously described in the preconditioning phenomena like heat shock proteins (6, 52), receptor of protein tyrosine kinase activity (53, 54), and the response to estradiol (55).

HRG1-alpha is an alternative spliced variant of *NEUROGULIN 1* (NRG1) gene, which is a glycoprotein that interacts with the ERBB2 receptor tyrosine kinase to increase its phosphorylation on tyrosine residues. There is a lack of literature on HRG1-alpha splice variant. However, NRG1 is related with synaptic plasticity and neuroinflammation (56), nerve repair (57), peripheral nervous system development (58). Neuroprotective and anti-inflammatory effects of NRG-1 are associated with the differential regulation of NF-kB signaling pathways in microglia (59, 60). *MAC-1* gene encodes the integrin alpha M chain. Integrins are heterodimeric integral membrane proteins composed of an alpha chain and a beta chain. Activation of the integrin MAC-1 is known to be critical for mediating neutrophil adhesion and migration. The expression of Mac-1 in microglia is heterogeneous both under normal conditions and after stroke (61).

## Limitations

The main limitation of the current study is the available TIA patients in our cohort [TIA patients = 8.1%], which have lead us to use a pooling method of sample processing in the inflammation antibody arrays, instead of the individual sample processing. As in other human diseases, the pathophysiological pathway of AIS is complex and heterogeneous. In order to confirm our findings, it would be beneficial to complete the study by single protein expression determination of potential candidate biomarkers in individual plasma samples in a larger cohort. Information of previous TIA among aphasic or severe AIS is difficult to obtain, although close familiars and caregivers were asked about it, some information might be lacking.

## Conclusions

Previous recent TIA before AIS is associated with better clinical and imaging outcomes than AIS without previous TIA. This fact could be explained by the induction of ischemic tolerance phenomena that is related to an inflammatory plasma cytokine signature. The determination of previous described biomarkers like IL-6 and S100b confirmed a specific inflammation signature of ischemic tolerance. Interestingly, the antibody array technology emerged as a plausible strategy to identify the effect to the inflammatory response related to brain ischemia and new biomarkers or targets for neuroprotection. However, our results need to be confirmed in larger cohorts and by other techniques. Finally, the etiology and the infarction size of TIA groups should be taken into account and its effects on the final results dissected.

## Data Availability Statement

The raw data supporting the conclusions of this article will be made available by the authors, without undue reservation.

## Ethics Statement

The studies involving human participants were reviewed and approved by Comité ètica de l'Hospital Universitari Arnau de Vilanova de Lleida. The patients/participants provided their written informed consent to participate in this study.

## Author Contributions

FP conceived the study and procured funding. LC-C, JF, and FP designed experiments. GM-C, JM-S, NA, ÁO, JR, ST, MG-C, JM-F, AC-C, and TS patients' recruitment and clinical data acquisition. LC-C, JF, GA, and FP sample processing and data analysis. GA and FP wrote the paper. All authors commented on and approved submission of this manuscript.

## Conflict of Interest

The authors declare that the research was conducted in the absence of any commercial or financial relationships that could be construed as a potential conflict of interest.
